# A knotted ureteral stent: A case report and review of the literature

**DOI:** 10.4103/0974-7796.65108

**Published:** 2010

**Authors:** Stefano Picozzi, Luca Carmignani

**Affiliations:** Department of Urology, IRCCS Policlinico San Donato, University of Milan, San Donato Milanese, Milan, Italy

**Keywords:** Ureter, ureteral stent, knotted ureteral stent, kidney stone, urological complication

## Abstract

The indications for ureteral stent placement have expanded significantly with the progress of surgical procedures and techniques. Although such stents are now an integral part of contemporary urological practice, their use is not free of complications and consequences. There are very rare descriptions of knot formation in a ureteral stent and the consequences of this occurrence, with only 12 cases previously reported. Here, we report an additional case and review all the literature concerning this urological complication with emphasis on its predisposing factors and conservative and surgical management.

## INTRODUCTION

Modern indwelling ureteral stents were first described by Zimskind *et al*. in 1967.[1]Since then, the indications for ureteral stent placement have expanded significantly with the progress of surgical procedures and techniques and such stents have become an integral part of contemporary urological practice.

Despite substantial advances, the use of ureteral stents is not free of complications and consequences. There are very rare descriptions of the knot formation in a ureteral stent and the consequences of this occurrence.

## CASE REPORT

A 41-year-old woman underwent combined urological and gynaecological surgery for deep pelvic endometriosis (stage 4 according to the American Fertility Society Classification[2]) with involvement of the lower third of the right ureter. Segmental ureterectomy was necessary and in order to repair this distal ureteral injury, a psoas bladder hitch with ureteroneocystostomy was performed using the Politano-Leadbetter technique with creation of a sub-mucosal tunnel. A 7 Fr 26 cm double-J stent was placed intra-operative under fluoroscopic guidance. The patient's post-operative recovery was uneventful and she discharged from hospital 4 days after the surgical procedure.

**Figure 1:**
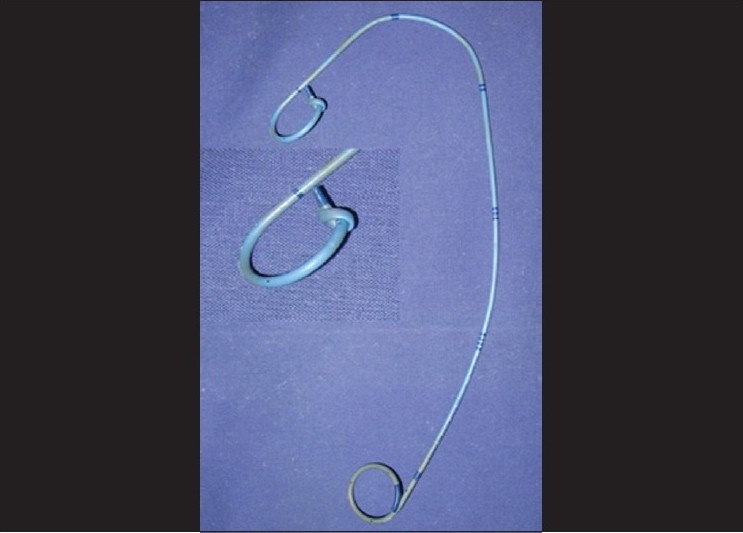
A complete knot is present in the proximal portion of the indwelling double-J ureteral stent

Thirty days after her operation, the patient came to our Urology Department for remove of the ureteral stent. Slight difficulty in extraction of the stent was noted by the operator, but with continuous mild traction it was removed completely. Examination of the ureteral catheter revealed a knot in its proximal J [Figure 1]. An endoscopic evaluation was carried out to exclude any damage to the neo-anastomosis or bleeding from the ureter and the ureteroneocystostomy site. After observation, the patient was discharged with anti-oedema therapy, corticosteroids and antibiotics. Eight hours after the procedure, a renal colic was resolved with analgesic drugs and the patient remained asymptomatic thereafter. Careful follow-up based on sonography of the upper urinary tract and urography at three months, associated with clinical evaluations, did not reveal any complications.

## DISCUSSION

The complications and consequences of the use of indwelling ureteral stents include bladder storage and irritative voiding symptoms, flank and/or suprapubic pain, stent colic, vesicorenal reflux, malposition, haematuria, urinary tract infection, bacteriuria, fever, encrustation, inadequate relief of obstruction, stent migration, stent rupture, ureteral perforation, erosion and fistulisation.

One other complication is knotting of the stent. Since the first report in 1989, only twelve cases have been published in the international literature [Table 1].[2-13] The patients' ages ranged between 4 to 86 years with a male to female ratio of 7 to 4. The knots were reported in the upper end (ten cases), lower end (one case) and ureteral portion (one case).

**Table 1:** Resume of all cases reported in literature

Authors	Age and gender	Pathology	Catheter configuration	Diameter	Location of the knot	Treatment for stent removal
Groeneveld AE, 1989[3]	NR	Renal stone	Double-j stent	NR	Proximal	Traction
Das G, Wickham JE, 1990[4]	45 M	Renal stone	Single-j ureteral catheter	NR	Distal	Traction
Braslis KG, Joyce G, 1992[5]	37 F	Renal stone	Multilength ureteral catheter	4.7 ch	Proximal	Percutaneous stent removal
Kundargi P *et al*., 1994[6]	53 M	Renal stone	Multi-coil ureteral catheter	6 f	Proximal	Percutaneous stent removal
Flam TA *et al*., 1995[7]	86 M	Upper ureteral stone	Circumflex double pig tail stent	6 f	Proximal	Placement of a double-j stent and one week later the knot was untied by grasping the upper tip of the stent via ureteroscopy
Baldwinn DD *et al*., 1998[8]	73 M	Maintain ureteroscopic access during surveillance ureteroscopies for transitional cell carcinoma	Multilength ureteral catheter	7 f	Proximal	Knot was untied with an amplatz super stiff guide wire passed retrograde via the ureteral stent
Quek M, Dunn MD, 2002[9]	66 F	Ureteropelvic junction stone	Double-j stent	7 f	Mid	Traction
Sighinolfi MC *et al*., 2005[10]	48 M	Renal stone	Multilength ureteral catheter	5 ch	Proximal	Three days of continuous slight traction
Kondo N *et al*., 2005[11]	37 M	Renal stone	Multilength ureteral catheter	6 f	Proximal	Ureterotomy
Corbett HJ, Dickson AP, 2005[12]	4 M	Reimplantation of an obstructed mega-ureter	Multilength ureteral catheter	4.7 f	Proximal	Traction
Eisner B *et al*., 2006[13]	82 F	Renal stone	Multilength ureteral catheter	6 f	Proximal	Traction

The majority of the cases involved multilength coil stents (8/12 cases). These ureteral catheters, designed to meet the "one size fits all" concept, are associated with a lower risk of migration but appear to have a higher risk of knotting compared to double-J and single-J stents; the reason may lie in the substantial length of tubing that remains in the kidney to form the multi-coil configuration associated with the configuration of the dilatated renal pelvis or the presence of a stone that could potentially influence coil configuration during stent positioning. As regards double-J stents, stent migration with subsequent excess catheter length was described in two cases.[6,8] In our case the stent length was excessive because of the segmental ureterectomy and bladder psoas hitch. For double-J stents, the excess of tubing length in one of the extremities could, therefore, be considered the main factor contributing to knotting. In the only case of knotting of a single-J stent the excess of tubing length is the main contributing factor.

The diameters of the stents that knotted varied from 4.7 ch to 7 ch and were equally distributed, so thinner stents with their greater flexibility do not appear to predispose to this complication.

In all reported cases, the knot in the upper and ureteral portion was detected by X-ray only after cystoscopic removal of the stent and there are no reports of knot formation previously documented by radiographs when performed. We can, therefore, postulate that modifications of coil configuration can lead to the creation of knots during traction.

If resistance is noted during stent traction, the manoeuvre should be stopped to avoid ureteral damage. Immediate X-ray studies revealed the underlying complication in 7 of 8 cases in which they were performed, while in one case the knot was interpreted as an encrustation.[9]

Various techniques have been used to deal with knotting of the upper or middle portions, including simple traction (6 cases), simple traction and delayed removal (1 case), removal with guide wire assistance (1 case), ureteroscopy (1 case), ureterotomy (1 case), and percutaneous removal (2 cases).

In one case Basavaraj *et al*. performed simple traction and delayed removal after having successfully left the stent *in situ* with the intention that the stretch knot would slowly dilate the ureteral stricture localized in the impacted area of the ureteroileal anastomosis.[13] In two cases the stent was removed after that a tight knot had been untied: Baldwin *et al*. passed an Amplatz 0.038 Super Stiff guide wire from the proximal tip of the stent to untie the knot and Flam *et al*. placed a second ureteral stent alongside the knotted stent and a week later untied the stent during ureteroscopy with 5F alligator forceps.[6,7]

The risk of dangerous ureteral trauma or avulsion must be considered when applying graduated traction. All surgeons stopped traction if unusually strong resistance was noted and complications of traction were reported in only one patient who developed abdominal pain and hydronephroureter; these symptoms resolved within 48 hours.[11] Our patient developed renal colic, successfully treated with analgesic drugs, 8 hours after the extraction procedure.[14]

Surgical removal of knotted stents, by ureterotomy and percutaneous access, was performed only after a conservative technique had failed.
